# Routes of Allergic Sensitization and Myeloid Cell IKKβ Differentially Regulate Antibody Responses and Allergic Airway Inflammation in Male and Female Mice

**DOI:** 10.1371/journal.pone.0092307

**Published:** 2014-03-25

**Authors:** Astrid Bonnegarde-Bernard, Junbae Jee, Michael J. Fial, Haley Steiner, Stephanie DiBartola, Ian C. Davis, Estelle Cormet-Boyaka, Daniel Tomé, Prosper N. Boyaka

**Affiliations:** 1 Department of Veterinary Biosciences, The Ohio State University, Columbus, Ohio, United States of America; 2 Laboratory of Human Nutrition, AgroParisTech, Paris, France; 3 Department of Internal Medicine, The Ohio State University, Columbus, Ohio, United States of America; French National Centre for Scientific Research, France

## Abstract

Gender influences the incidence and/or the severity of several diseases and evidence suggests a higher rate of allergy and asthma among women. Most experimental models of allergy use mice sensitized via the parenteral route despite the fact that the mucosal tissues of the gastrointestinal and respiratory tracts are major sites of allergic sensitization and/or allergic responses. We analyzed allergen-specific Ab responses in mice sensitized either by gavage or intraperitoneal injection of ovalbumin together with cholera toxin as adjuvant, as well as allergic inflammation and lung functions following subsequent nasal challenge with the allergen. Female mice sensitized intraperitoneally exhibited higher levels of serum IgE than their male counterparts. After nasal allergen challenge, these female mice expressed higher Th2 responses and associated inflammation in the lung than males. On the other hand, male and female mice sensitized orally developed the same levels of allergen-specific Ab responses and similar levels of lung inflammation after allergen challenge. Interestingly, the difference in allergen-specific Ab responses between male and female mice sensitized by the intraperitoneal route was abolished in IKKβ^ΔMye^ mice, which lack IKKβ in myeloid cells. In summary, the oral or systemic route of allergic sensitization and IKKβ signaling in myeloid cells regulate how the gender influences allergen-specific responses and lung allergic inflammation.

## Introduction

Compelling evidence suggests that gender differences exist in the severity of many immune diseases, including inflammatory diseases [1] such as allergy and asthma [2]. Accordingly, males have a higher risk of bacterial and viral infections [3], whereas women are more susceptible to autoimmune and inflammatory diseases [4]. Allergic asthma is an inflammatory disease of the airways characterized by airway hyper-responsiveness, airway inflammation with recruitment of lymphocytes and eosinophils, as well as mucus cell hyperplasia [5]. Allergic asthma has been reported to differentially affect men and women, and gender-based differences were described between children *vs* adult or before *vs* after puberty [6], [7]. For example, boys have a higher prevalence of allergy and asthma compared with girls but the proportions are reversed after puberty [8]. Despite clinical evidence, the reasons behind this gender difference are not well understood. The effect of hormones is unclear and studies have not been conclusive either in human or in animal models [9]. Some studies have shown that androgens could have a protective effect in males whereas estrogen could have deleterious effects on airway inflammation in females [9].

The pathophysiological and immunological characteristics of allergic immune responses are controlled by a variety of factors. Thus, the magnitude and quality of the allergen-specific immune response are controlled by the dose and frequency of exposure to allergen, the type of adjuvant, the genetic background, the route of sensitization and the gender. Previous studies showed that the subcutaneous route was more effective than the intraperitoneal (ip) route at inducing IgE antibodies [10]. Allergic sensitization to ingested antigens can be modeled by administration of antigens together with an adjuvant capable of breaking oral tolerance, such as cholera toxin [11]–[13] or the B subunit of Staphylococcus enterotoxin (SEB) [14], [15]. In this regard, we have previously shown that allergic inflammation can develop in the airways following nasal allergen challenge of mice sensitized by the oral route [12], [13].

Cytokines produced by myeloid cell subsets, including macrophages and dendritic cells, influence differentiation of naive T lymphocytes into Th2 cells capable of supporting induction of IgE Abs and effector cells involved in allergic responses. The nuclear factor κB (NF-κB) pathway plays an important role in cytokine responses to a variety of stimuli [16]. Its activation is regulated by the IκB kinase (IKKβ), the catalytic subunit of the IKK complex responsible for NF-κB translocation and transcription [16]. IKKβ-NF-κB signaling controls a number of biological processes via tissue-specific regulation of inflammatory and anti-inflammatory responses and can mediate both pro- and anti-inflammatory effects [17], [18]. It has been suggested that an increased expression of interleukin-6 in monocytes in women contributes to gender differences in the risk of inflammatory disorders [19]. However, how and to what extent NF-κB regulates allergic responses in males and females remain poorly understood.

Experimental murine models of allergy and asthma use mostly female mice and the possible influence of gender on allergen-induced lung inflammation has not been thoroughly investigated. To address this question, we compared allergen-specific Ab responses and subsequent responses to nasal allergen challenge in male and female mice sensitized either orally or via ip injection with the model food antigen ovalbumin (OVA) and cholera toxin as adjuvant. We show that ip sensitization of adult mice results in a significant gender-disparity with female mice developing higher IgE and Th2-associated response and allergic airway inflammatory responses upon allergen challenge. Such differences were not seen in male and female mice sensitized via the gastrointestinal tract, suggesting that gender differentially influences allergic sensitization depending on the route of exposure.

## Materials and Methods

### Mice and Ethic Statement

Wild-type C57BL/6 mice were obtained from the Jackson Laboratories (Bar Harbor, ME) or NCI (Frederick, MD) and acclimated in our facility before use. The IKKβ^ΔMye^ mice, in which IKKβ-dependent NFκB signaling was eliminated in myeloid cells [17], were bred in our facility. All mice were maintained in a pathogen-free environment and used at 8–12 wks of age. Studies were approved by the Ohio State University Institutional Animal Care and Use Committee (Protocol number: 2008A0210R) and were performed in accordance with both National Institutes of Health and Ohio State University Institutional Animal Care and Use Committee guidelines to avoid pain and distress.

### Histology

Immunohistochemistry was performed on 5 μm sections of formalin-fixed and paraffin-embedded tissues. Lung tissues were inflated before fixation and tissue sections were stained with hematoxylin and eosin according to standard protocols. Quantification of lung inflammation was performed in a blinded fashion by two independent investigators as previously described [11].

### Intraperitoneal or Oral Sensitization and Nasal Challenge of Mice

Oral sensitization to the food antigen was performed on days 0 and 7 by intragastric administration of 250 μl of PBS containing 1 mg of ovalbumin (OVA) and 10 μg of cholera toxin as adjuvant. Parenteral sensitization was performed on days 0 and 7 by intraperitoneal injection of 100 μl of PBS containing 100 μg of OVA and 1 μg of cholera toxin. Nasal antigen challenges were performed on days 15, 16 and 19 with 100 μg of OVA as previously described [11].

### ELISA for Antigen-specific Ab Responses

Plasma samples were collected one week after each sensitization, on days 7 and 14, and plasma levels of OVA-specific antibodies were measured by enzyme-linked immunosorbent assay (ELISA) as previously described [11].

### Analysis of Lung Functions and Airway Responses to Methacholine Challenge

Mechanical properties of the mouse lung were assessed on anesthetized mice using the forced-oscillation technique as previously described [11]. Mice were exposed to increasing doses of methacholine (0.1, 1, 10, 20 and 50 mg/ml) and ten recordings of total lung resistance were generated following administration of each methacholine dose [11], [12].

### Real-Time RT-PCR Analysis of mRNA Responses

Cytokine mRNA responses antibodies were analyzed by Real-Time RT-PCR as previously described [11].

### Statistics

Results are expressed as the mean ±1 SD. Unless otherwise indicated, statistical significance was determined by Student’s T test or by ANOVA followed by the Fisher Least Significant Difference Test (Statistica software package, StatSoft, Inc., Tulsa, OK). For statistical analysis, antibody levels below the detection limit were recorded as two log_2_ below the detection limit. All tests were considered significant at a probability of *p*<0.05.

## Results

### Gender Influences Allergen-specific Ab Responses to Parenteral but not Oral Sensitization

**Figure 1:**
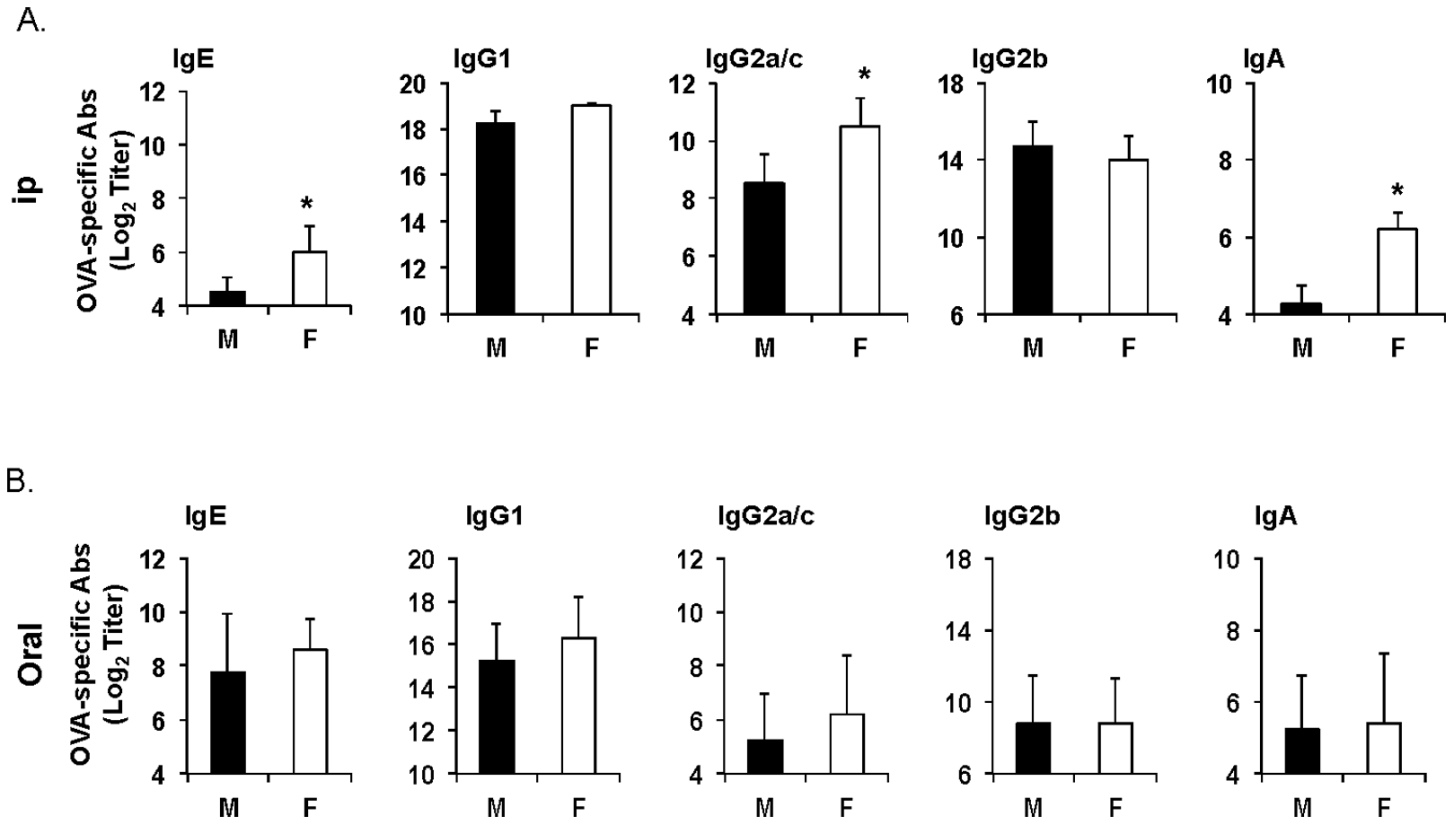
Gender does not alter the pattern of antigen-specific serum Ab responses to ingested antigens. (**A**) Male (black) and female (white) mice were sensitized on days 0 and 7 by intraperitoneal injection of ovalumin (0.1 mg) and cholera toxin (1 μg) (**B**) Mice were sensitized by oral administration of ovalbumin (1 mg) and cholera toxin (10 μg). Blood was collected on day 14. OVA-specific IgE, IgA isotypes and IgG subclasses (i.e., IgG1, IgG2a/c, IgG2b and IgG3) were measured by ELISA. The results are expressed as the log_2_ titers ± one SD from three experiments with five mice per group.

We first examined the antibody responses after oral or parenteral (i.e., intraperitoneal) sensitization. In mice sensitized by the ip route, the IgE, IgG2a/c levels were higher in females (Fig. 1A). In contrast, male and female mice sensitized by the oral route showed no differences in their level of Th1- (i.e., IgG2a/c) or Th2-associated (i.e., IgE, IgG1) antibodies (Fig. 1B). Consistent with the known ability of cholera toxin as an oral adjuvant to induce generalized IgA antibody responses, mice developed antigen-specific serum IgA responses after oral sensitization and similar levels of IgA response were seen in male and female mice. Mice sensitized by the parenteral intraperitoneal (ip) route also developed IgA responses and again, females exhibited higher levels of IgA responses (Fig. 1).

### Gender Significantly Affects Allergic Airway Inflammation in mice Sensitized via the Parenteral Route

**Figure 2:**
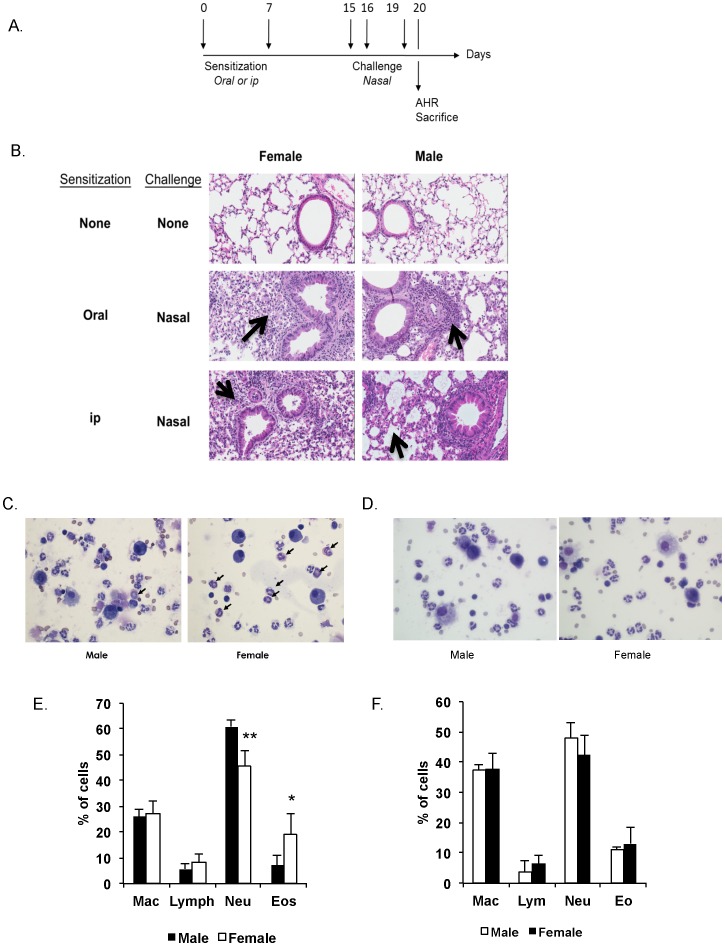
Gender influences lung inflammation after nasal antigen challenge in mice sensitized by the intraperitoneal route. Mice were sensitized on days 0 and 7 either by oral administration of ovalbumin (1000 μg) and cholera toxin (10 μg) or intraperitoneal injection of ovalbumin (100 μg) and cholera toxin (1 μg). Nasal challenges were performed on days 15, 16 and 19 and lungs were collected on day 20. (**A**) Timeline of experiments showing the days of intervention. (**B**) Hematoxylin and eosin staining of lung sections from C57BL/6 mice (magnification×200). Black arrows denote areas of inflammation. The results are representative of three experiments with five mice per group. (**C**) Pictures of BAL in mice immunized ip or orally with OVA and nasally challenged (×600). Black arrows denote eosinophils. (**D**) Percentage of immune cell populations in BALF in mice sensitized orally or intraperitoneally and nasally challenged. Results are expressed as mean ± SD of 5 mice per group. (*, p<0.05).

We next examined whether the gender-induced difference in allergen-specific Ab responses would affect responses to nasal antigen challenge (Fig. 2A). Histology showed no difference between lung tissues of naïve male and female mice, and mice not sensitized but challenged displayed no sign of inflammation (data not shown). Female mice nasally challenged after ip sensitization exhibited a broader inflammatory response than males, with cell recruitment into the lung parenchyma and in the perialveolar spaces (Fig. 2B). After nasal challenge, no gender difference was seen in the phenotype of immune cells in the BAL of orally sensitized mice (Fig. 2D, 2F). The same number of cells was collected in BAL of mice sensitized by the ip route, but the females expressed lower numbers of neutrophils and higher numbers of eosinophils than males (Fig. 2C and 2E).

### Airway Hyper-responsiveness does not Mirror Gender Difference in Lung Inflammatory Responses of mice Sensitized by the Parenteral Route

**Figure 3:**
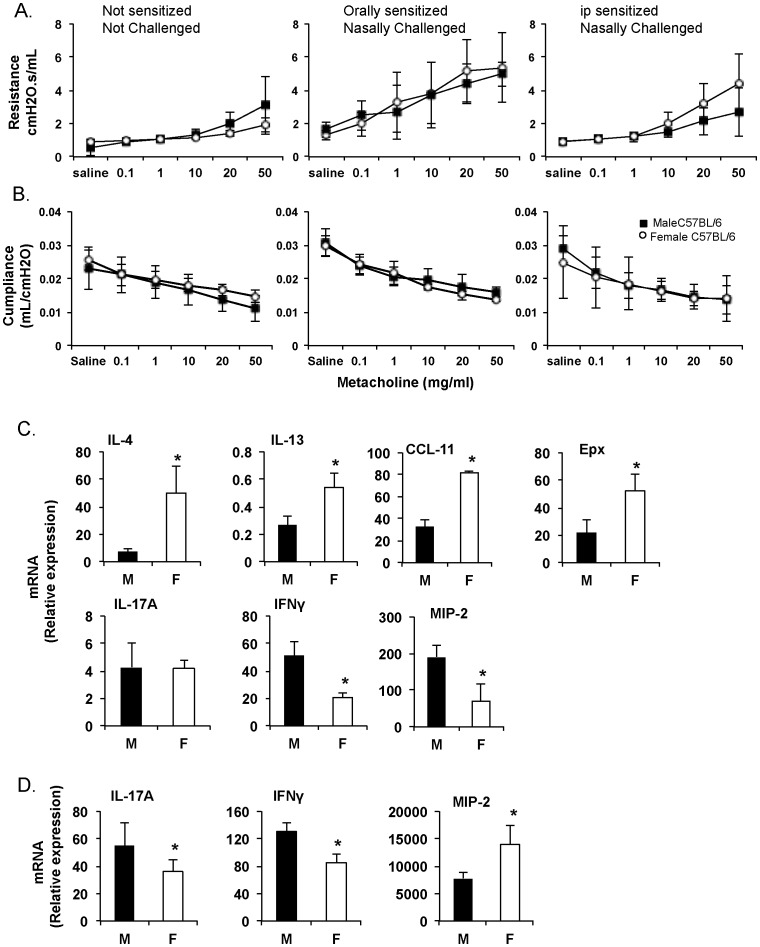
Airway hyper-responsiveness and cytokine responses to nasal challenge of male and female mice sensitized by the oral or parenteral route. Mice were sensitized on days 0 and 7 either by oral administration of ovalbumin (1000 μg) and cholera toxin (10 μg) or intraperitoneal injection of ovalbumin (100 μg) and cholera toxin (1 μg). (**A**) Mice were exposed to increasing amounts of methacholine and lung resistance was measured. (**B**) Mice were exposed to increasing amount of methacholine and lung compliance was measured. (**C**) Real-time RT-PCR analysis of cytokine, chemokine and eosinophil peroxidase mRNA responses in lung tissues after nasal antigen challenge in mice immunized ip with OVA and CT. (**D**) Cytokine, chemokine and eosinophil peroxidase mRNA responses in lung tissues after nasal antigen challenge in mice immunized orally with OVA and CT. Results are expressed in Relative Copy Number (1/2∧ΔCt *100*1000) as mean ± SD of three separate experiments, with 5 mice per group. (* p<0.05).

Allergen-induced airway hyper-responsiveness (AHR) is an important outcome of inflammation and airway remodeling in asthma. Twenty-four hours after the last challenge with OVA (Fig. 2A), there was a significant increase in airway reactivity to methacholine in previously sensitized mice (Fig. 3A, 3B). Male and female mice sensitized by the oral route exhibited similar lung function characteristics and responses to methacholine challenge. Interestingly, gender did not significantly influence airway resistance in mice sensitized by the ip route; although females tended to express higher lung resistance (Fig. 3A). Furthermore, the pulmonary compliance (changes in lung volume for any given applied pressure) in challenged mice was the same regardless of gender (Fig. 3B).

Airway hyper-responsiveness also is controlled by cytokines secreted in response to antigen challenge; we and others have previously shown that differences in cytokine responses can lead to discrepancies between lung inflammation and AHR [11], [12]. Nasal antigen challenge of ip-sensitized female mice promoted higher levels of eotoxin (CCL11) and eosinophil peroxidase (Epx), and lower levels of MIP-2 mRNA responses in the lungs than in males (Fig. 3C), consistent with the cell profile in BALF. On the other hand, orally sensitized female mice exhibited higher MIP2 but reduced IL-17A and IFN-γ mRNA responses than males (Fig. 3D). Th2-related cytokine (i.e., IL-4, IL-13, CCL-11) mRNA responses were not different between male and female mice that were sensitized orally (not shown).

### IKKβ in Myeloid Cells Regulates Responses of Male and Female mice to Allergic Sensitization via the Parenteral Route

**Figure 4:**
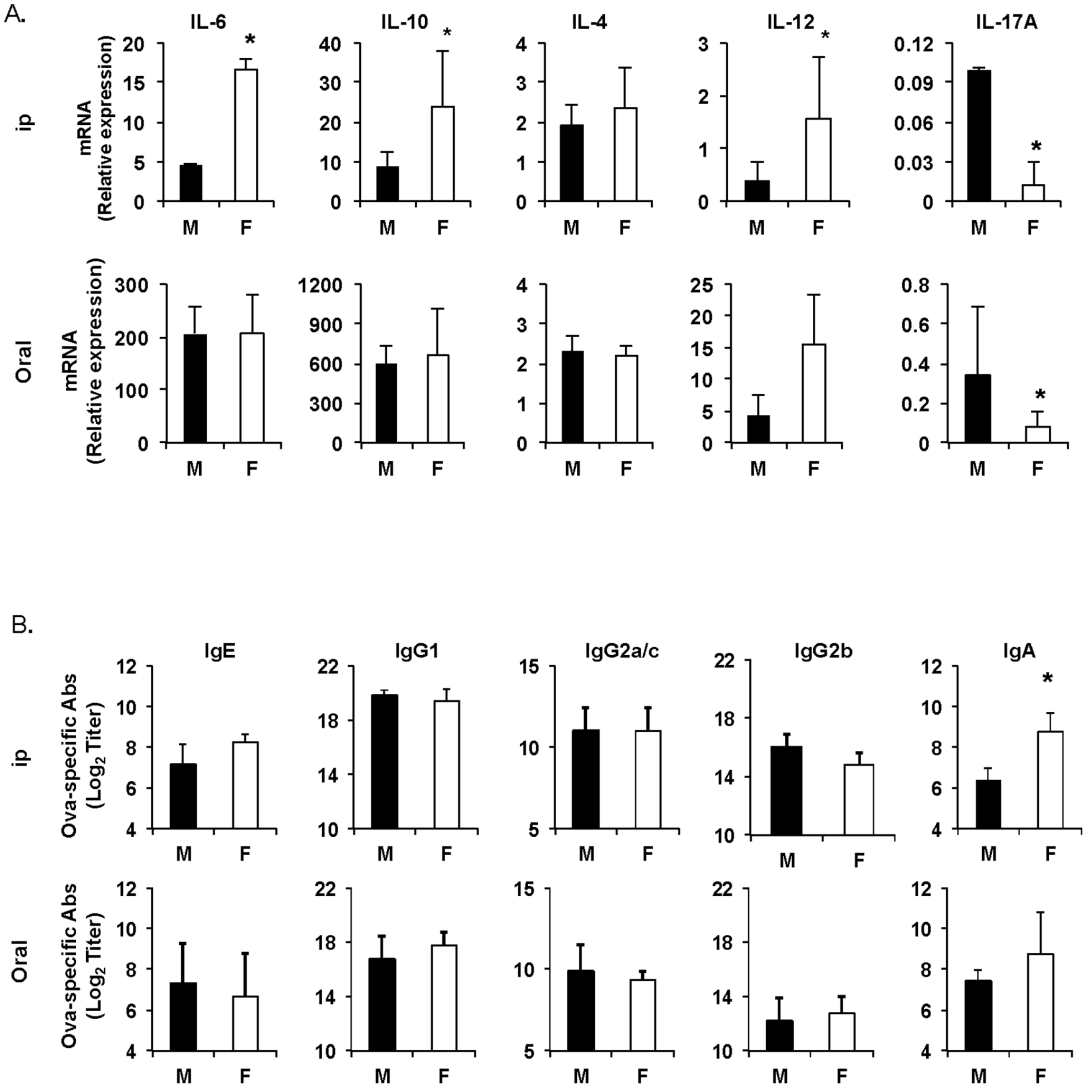
IKKβ in myeloid cells regulates male and female responses to intraperitoneal sensitization to allergen. (**A**) Cytokine responses in spleen tissues after oral or intraperitoneal sensitization. Spleens were collected 16 hours after intraperitoneal or oral sensitization and cytokine mRNA responses were determined by Real-time RT-PCR. (**B**) IKKβ^ΔMye^ mice were sensitized on days 0 and 7 by intraperitoneal injection of ovalbumin (100 μg) and cholera toxin (1 μg) or by oral administration of ovalbumin (1 mg) and cholera toxin (10 μg). Blood was collected on day 14. OVA-specific IgE, IgA isotypes and IgG subclass were measured by ELISA. The results are expressed as the log_2_ titers ± one SD of two experiments with five mice per group.

Myeloid cells are major contributors of pro-inflammatory cytokines. As mentioned above, it has been suggested that an increased expression of interleukin-6 by monocytes in women could contribute to the higher risks of inflammatory disorders in this population when compared to men [19]. To test whether these cells could also play a role in the gender difference with regard to responses to allergic sensitization, we first measured mRNA cytokine responses in the spleens 16 hours after oral or parenteral administration of cholera toxin and OVA. Female mice sensitized by the ip route clearly showed higher IL-6, IL-10 and IL-12 mRNA responses than males (fig. 4A). On the other hand, female and male mice sensitized by the oral route exhibited similar levels of cytokine mRNA responses (Fig. 4A). We next analyzed allergen-specific serum antibody responses after oral or parenteral sensitization of mice lacking IKKβ in myeloid cells (IKKβ^ΔMye^ mice). Interestingly, no difference was found in the level of serum IgG and IgE antibodies between male and female IKKβ^ΔMye^ mice sensitized by the parenteral route, except for IgA, which was higher in females (Fig. 4B). As with wild-type mice (Fig. 1B), male and female IKKβ^ΔMye^ mice sensitized by the oral route exhibited no difference in antibody responses (Fig. 4B).

**Figure 5:**
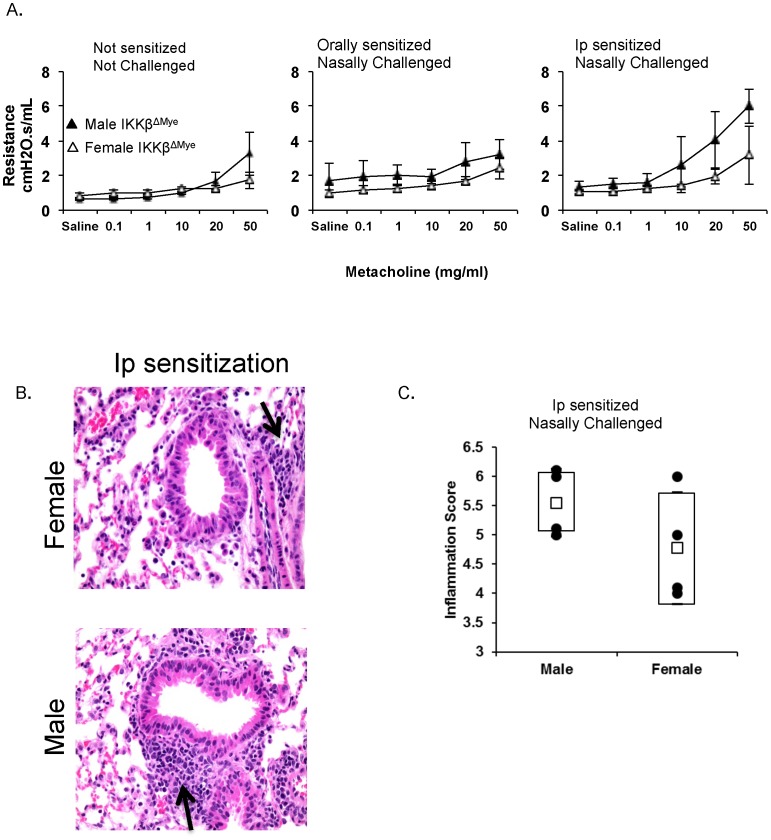
Airway hyper-responsiveness to nasal challenge and lung inflammation in male and female IKKβ^ΔMye^ mice sensitized by the oral or parenteral route. The IKKβ^ΔMye^ mice were sensitized on days 0 and 7 either by oral administration of ovalbumin (1000 μg) and cholera toxin (10 μg) or intraperitoneal injection of ovalbumin (100 μg) and cholera toxin (1 µ g). (**A**) Mice were exposed to increasing amounts of methacholine and lung resistance was measured. The results are expressed as mean resistance ± one SD (five mice per group). (**B**) Hematoxylin and eosin staining of lung sections from male and female IKKβ^ΔMye^ mice sensitized by intraperitoneal injection (magnification×200). The results are representative of two experiments with four mice per group. (**C**) Inflammation scores after nasal antigen challenge of male and female IKKβ^ΔMye^ mice sensitized by intraperitoneal injection (four mice per group).

We next examined whether the changes in allergen-specific responses induced by IKKβ deficiency in mice sensitized by the parental route also affect allergen-induced airway hyper-responsiveness. Twenty-four hours after the last antigen challenge, both male and female IKKβ^ΔMye^ mice sensitized orally failed to develop airway reactivity to methacholine (Fig. 5A). Female IKKβ^ΔMye^ mice sensitized via the parenteral route also failed to show increased lung resistance to methacholine challenge. Surprisingly, male IKKβ^ΔMye^ mice sensitized via the parenteral route retained the ability to develop allergen-induced airway hyper-responsiveness, suggesting that the IKKβ pathway plays a major role in allergen-induced airway hyper-responsiveness in female mice. This discrepancy in lung responses between male and female IKKβ^ΔMye^ mice sensitized via the parenteral route was further confirmed by the analysis of lung inflammation (Fig. 5B and 5C). Thus, lungs of male IKKβ^ΔMye^ mice showed higher levels of inflammation than those of female IKKβ^ΔMye^ mice sensitized via the same parenteral route (Fig. 5B and 5C).

## Discussion

Experimental and clinical data indicate a difference in gender susceptibility to airway hyper-responsiveness and allergic inflammation. However, these differences appear to vary with the experimental system used and the end point analyzed. There are known differences in lung airway size, alveolar number and volume between males and females [20]. Thus, gender disparities exist in the architecture of the lungs between males and females, where naive female mice have 50% smaller alveoli than male mice [20]. Even though the terminal gas-exchange units are reduced in size in female mice, this does not affect the transport of oxygen or lung function. The main objectives of this study were to investigate the influence of gender in the development of airway inflammation and hyper-responsiveness in a murine model of allergic lung inflammation and to study whether the route of allergic sensitization affects these responses.

Several mouse strains have been studied to characterize pulmonary functions [21]. The C3H/HeJ and BALB/c mice are recommended for mutagenesis research because of their sensitivity to chemicals whereas A/J and C57BL/6 mice can serve as animal models to study allergic airway diseases because of their divergent airway responsiveness. Other comparison studies have shown that C3H/HeJ have the highest static compliance values, while C57BL/6 have the lowest [22]. The A/J mice tend to develop a more severe form of asthma while C3H/HeJ are more prone to develop mild asthmatic disease [22]. Furthermore, BALB/c mice, which develop strong Th2 responses, have been used extensively to study allergic responses and asthma after parenteral sensitization with the adjuvant alum [23], [24]. In this report, we have used C57BL/6 mice to investigate whether gender can influence the development of inflammatory responses in the airways. The relevance of determining the gender difference between male/female in C57BL/6 lies in the ability of these mice to develop Th2 responses and IgE after oral immunization with cholera toxin as adjuvant [25]. It is important to indicate that alum, which has been widely injected into mice by the intraperitoneal to induce allergic type responses, is not an effective adjuvant when given by the oral route.

Airway hyper-responsiveness to cholinergic agents like methacholine is a defining feature in experimental asthma. Furthermore, airway hyper-responsiveness in the context of allergic asthma generally is associated with allergic inflammation characterized by eosinophilia and mucus production [22]. Based on changes in resistance, elastance and other mechanical parameters, other studies have demonstrated that female mice are more sensitive than males to inhaled methacholine [26]–[28]. Accordingly, we showed that females immunized intraperitoneally with OVA and cholera toxin develop higher IgE levels and broader lung inflammation. It important to note that similar results were recently reported in a study where male and female mice were sensitized by intraperitoneal injection of OVA with alum as adjuvant [29]. Thus, the difference in responses between males and females was not due the pharmacological effect of the cholera toxin adjuvant in our study. Interestingly, after nasal challenge, male and female mice sensitized intraperitoneally with OVA and cholera toxin showed slight differences in airway hyper-responsiveness, but only with high doses of methacholine and without reaching statistical significance. It is important to indicate that we used an invasive constant phase method (Flexivent) for the analysis of airway responsiveness whereas other studies have used unrestrained plethysmography (Penh). In this regard, the reliability of Penh for evaluation of airway responsiveness was recently questioned [30], and the suggestion was made to limit its use to the measure of patterns of respiration. Thus, despite its simplicity and non-invasive advantage, Penh measurement suffers from biased parameters, like natural breathing, which alter the measurements.

Most murine models of asthma evaluate responses after intraperitoneal sensitization followed by several nasal challenges. The oral route of sensitization we used in this study more likely recapitulates events that occur during common sensitization to food allergens. The importance of the route of sensitization has been already demonstrated by Hansen et al. who compared ip versus nasal sensitization [8]. We show that oral immunization does not discriminate between males versus females and both develop equal levels of allergen-specific Ab responses and AHR. We did not find differences in the level of Th2 antibody responses (IgE and IgG1) between male and female mice immunized orally. This finding is in contrast with previous studies by others who showed higher serum IgE and IgG1 levels in female mice sensitized by the intraperitoneal route with alum as adjuvant [28], [31]. However, we found that female mice sensitized by the intraperitoneal route with cholera toxin as adjuvant also develop higher levels of allergen-specific IgE and IgG2a/c, as well as IgA Ab responses than males. Thus, the difference between the responses of male and female mice in these studies is primarily due to the route of sensitization rather than the adjuvants used.

The immune system in mucosal tissues of the GI tract has evolved alongside, but separate from the general bloodstream to adapt to the unique environment in this site. Mine et al. [32] have demonstrated a substantial heterogeneity in IgE-binding epitopes, between groups of BALB/c mice sensitized to OVA via three different routes of exposure, i.e., oral, subcutaneous and intraperitoneal routes. We now provide evidence that different patterns of innate cytokine responses are induced early in male and female mice but only after parenteral sensitization. The higher levels of IL-6, IL-10 and IL-12 mRNA seen in female mice shortly after parenteral administration of cholera toxin more likely contribute to the difference in allergen-specific responses developed by male and female mice. Thus, the fact that these differences are eliminated in IKKβ^ΔMye^ mice further supports the notion that myeloid cells and other innate cells at the site of allergic sensitization control the profile of allergen-specific B and T cell responses and subsequent pathologic responses to allergen challenge.

In summary, we have shown than allergic sensitization via the gastrointestinal tract leads to similar patterns of allergen-specific immune responses in male and female mice. We also showed that IKKβ in myeloid cells plays a key role in the gender disparity with regard to responses to intraperitoneal sensitization and further studies are warranted to better understand underlying mechanisms. Finally, our findings add support to the notion that airway hyper-responsiveness to methacholine challenge do not always reflect differences in airway inflammation. Thus, careful selection of experimental models and assays are crucial to study mechanisms of gender disparity for allergic diseases.
